# Associative Stimulation of the Supraorbital Nerve Fails to Induce Timing-Specific Plasticity in the Human Blink Reflex

**DOI:** 10.1371/journal.pone.0013602

**Published:** 2010-10-26

**Authors:** Kirsten E. Zeuner, Arne Knutzen, Asmaa Al-Ali, Mark Hallett, Günther Deuschl, Til O. Bergmann, Hartwig R. Siebner

**Affiliations:** 1 Department of Neurology, Christian-Albrechts-University Kiel, Kiel, Germany; 2 Human Motor Control Section, Medical Neurology Branch, National Institute of Neurological Disorders and Stroke, National Institutes of Health, Bethesda, Maryland, United States of America; 3 Danish Research Centre for Magnetic Resonance, Copenhagen University Hospital Hvidovre, Hvidovre, Denmark; 4 Institute of Neurology, Psychiatry and Senses, Medical Faculty, Copenhagen University, Copenhagen, Denmark; The Research Center of Neurobiology-Neurophysiology of Marseille, France

## Abstract

**Background:**

Associative high-frequency electrical stimulation (HFS) of the supraorbital nerve in five healthy individuals induced long-term potentiation (LTP)-like or depression (LTD)-like changes in the human blink reflex circuit according to the rules of spike timing-dependent plasticity (Mao and Evinger, 2001). HFS given at the onset of the R2 component of the blink reflex (HFS_LTP_) produced a lasting facilitation of the R2, whereas HFS given shortly before R2 (HFS_LTD_) caused a lasting suppression of the R2. In patients with benign essential blepharospasm (BEB), a focal dystonia affecting the orbicularis oculi muscles, HFS_LTP_ induced excessive LTP-like associative plasticity relative to healthy controls, which was normalized after botulinum toxin (BTX) injections (Quartarone et al, 2006).

**Methodology/Principal Findings:**

We used HFS conditioning of the supraorbital nerve to study homeostatic metaplasticity of the blink reflex circuit in healthy subjects and dystonic patients. On separate days, we tested the conditioning effects on the R2 response and paired-pulse R2 inhibition after (i) HFS_LTP_, (ii) HFS_LTP_ followed by HFS_LTP_, and (iii) HFS_LTP_ followed by HFS_LTD_. Controls also received (iv) HFS_LTD_ alone and (v) a non-intervention protocol. In BEB patients, HFS_LTP_ followed by HFS_LTD_ was given before and after BTX treatment. We were not able to replicate the bidirectional timing-dependent effects of HFS_LTP_ and HFS_LTD_ alone. All HFS protocols produced a non-specific reduction of the R2 response and a relative decrease in paired-pulse inhibition. These R2 changes also occurred in controls when no HFS was applied. There was also no trace of a homeostatic response pattern in BEB patients before or after BTX treatment.

**Conclusion/Significance:**

Our data challenge the efficacy of associative HFS to produce bidirectional plasticity in the human blink reflex circuit. The non-specific decrease of the R2 response might indicate habituation of the blink reflex following repeated electrical supraorbital stimulation. The increase of inhibition after paired pulse stimulation might reflect homeostatic behaviour to prevent further down regulation of the R2 response to preserve the protection of this adverse-effects reflex.

## Introduction

Synaptic plasticity refers to lasting changes in synaptic strength [1]. Synaptic strength is bidirectional modifiable by different patterns of presynaptic activity [1]. According to the learning rule introduced by Hebb [2], the synaptic connection between two neurons is strengthened if the firing of the presynaptic neuron is repeatedly and persistently paired with firing of the postsynaptic neuron. This idea has been refined in terms of temporal specificity [3] leading to the bidirectional rule of spike timing-dependent plasticity (STDP), which can be summarized as follows: synapses in which the presynaptic input precedes postsynaptic firing become strengthened (long-term potentiation (LTP)), whereas synapses in which presynaptic input follows postsynaptic firing become weakened (long term depression (LTD)) [4]. Synaptic plasticity is further controlled by homeostatic mechanisms which keep the shifts in synaptic strength within a physiological range [5]. Homeostatic metaplasticity adjusts the strength of synapses to prolonged changes in postsynaptic neural activity by dynamic modification of the thresholds for inducing LTP or LTD [1], [6]. Metaplasticity means synaptic plasticity of second order, i.e. plastic changes which alter the capacity of a given synapse to develop LTP or LTD [7]. Metaplasticity is often governed by homeostatic mechanisms that help to maintain synaptic strength within a functional range [8] This means that the capacity to undergo LTP or LTD is modulated by the recent history of synaptic activation in a homeostatic fashion introducing a bias towards LTP after prolonged inactivity and towards LTD after persistent activation. A sustained increase in postsynaptic neuronal activity lowers the threshold for inducing LTD while inhibiting the induction of LTP. Conversely, a reduction in postsynaptic neuronal activity decreases the threshold for inducing LTP whereas the induction of LTD becomes inhibited. The sliding modification threshold mediating homeostatic metaplasticity has been confirmed in *in vitro* and *in vivo* studies [5], [9]–[11]. This activity-dependency has been explicitly formulated in the Bienenstock-Cooper-Munro (BCM) model of bidirectional plasticity [6] and has gained substantial evidence in the field of motor learning [12].

The blink reflex is elicited with electrical stimulation of the supraorbital nerve with an early, ipsilateral R1 and a late, bilateral R2 response. In five healthy individuals, Mao and Evinger (2001) used associative high-frequency stimulation (HFS) of the supraorbital nerve to induce LTP- and LTD-like changes in the excitability of the trigeminal blink reflex circuit, as indicated by modulation of the R2 response [13]. When HFS was given at the onset of the R2 component of the blink reflex, HFS induced a lasting facilitation of subsequent R2 responses (HFS_LTP_). Conversely, HFS given shortly before R2 resulted in a lasting suppression of the R2 response (HFS_LTD_) [13]. Quartarone et al. (2006) replicated the LTP-like effects of HFS_LTP_ in 11 healthy subjects and showed an enhanced LTP-like facilitation of the R2 response after HFS_LTP_ in 16 patients with benign essential blepharospasm (BEB), a focal dystonia manifested by involuntary eyelid closure via the orbicularis oculi muscle [14]. This finding was interpreted as abnormal LTP-like associative plasticity in focal dystonia [15]. Moreover, botulinum toxin (BTX) treatment normalized the enhanced LTP-like plasticity of the blink reflex circuit [14]. By combining two interventional protocols, it is possible to probe homeostatic metaplasticity with transcranial stimulation techniques in the human motor cortex [16], [17]. Since homeostatic control of motor cortical plasticity is deficient in patients with focal hand dystonia [18], the present study was designed to assess homeostatic control of excitability in the human blink reflex circuit in healthy controls and patients with BEB.

We combined an inhibitory (HFS_LTD_) and facilitatory (HFS_LTP_) associative HFS protocol to test the following hypotheses: (i) The modification range of the blink reflex circuit, indicated by the R2 response and probed with associative HFS, is distorted in BEB with a stronger propensity towards LTP-like plasticity. (ii) Healthy subjects will show an occlusion of LTP-like plasticity and an increased tendency to develop LTD-like plasticity after pre-conditioning with HFS_LTP_. This homeostatic response pattern will be attenuated in BEB patients. (iii) Inhibitory HFS_LTD_ will induce a marked suppression (depotentiation) of the R2 response when given after HFS_LTP_. Patients with BEB will express no or less depotentiation because of deficient homeostatic plasticity. (iv) BTX treatment may transiently normalize abnormal plasticity patterns in BEB patients.

While previous studies only applied single electrical pulses to study changes in the unconditioned R2 response after HFS [13], [14], we added paired-pulse stimulation to quantify paired-pulse inhibition of R2 [19]. Previous studies reported defective paired-pulse R2 inhibition in BEB patients [19], [20] indicating enhanced excitability of brainstem interneuronal pathways [21] We reasoned that the relative loss of paired-pulse inhibition may correlate with abnormal plasticity responses to HFS conditioning in BEB patients.

## Materials and Methods

### Clinical data of patients

BEB patients (n = 16; 64±6 yr.; 9 female) and controls (n = 12; 50±14 yr.; 7 female) were included (Table 1). However, not all patients and controls participated in all protocols. Details are given in table 2 and 3. Patients and controls gave written informed consent to the protocol, which had been approved by the local ethics committee. Before each session, we evaluated location, influencing factors, severity of involuntary movements and disability using the Blepharospasm Rating Scale (BRS). One point is scored for each positive answer; the highest possible score is 40 points [22]. To evaluate blepharospasm clinically, each patient was evaluated according to the Blepharospasm Disability Scale (BDS) assessing the severity of dystonia in everyday life [22]. The BDS is described as 100%, meaning unaware of any difficulty; 95% with some blepharospasm, and 90% meaning socially affected. The scale uses a range of points from 0 to 5 for each of the 8 questions. For each patient, the total number of points scored was divided by the maximum possible points, the quotient multiplied by 90, and the result subtracted from 90%. The final score presents the percent of normal activity [22]. The lower the score the more is the patient clinically affected. We also recorded a 2-min video of spontaneous facial movements. A blinded examiner counted the number of blinks per minute at baseline and before the last block of measurement (after the HFS intervention).

**Table 1:** Clinical characteristics of patients.

Patient ID	Age (yr)	Symptom Duration (yr)	Clinical Symptoms	BRS points	BDS %	Number f blinks/min
P01	67	9	Cranial Dystonia	21	65.77	
P02	69	31	Cranial Dystonia	12	79.62	13
P03	51	7	Cranial Dystonia	9	79.62	52
P04	65	5	Blepharospasm	15	38.08	18
P05	65	13	Cranial Dystonia	17	20.77	17
P07	65	7	Blepharospasm	16	72.69	37
P08	66	9	Blepharospasm	7	79.62	34
P09	65	6	Blepharospasm	13	76.15	50
P10	57	7	Cranial dystonia	18	83.08	26
P11	72	16	Cranial Dystonia	10	83.08	25
P12	57	3	Blepharospasm	4	90.00	4
P13	63	7	Blepharospasm	8	90.00	39
P14	60	10	Blepharospasm	7	76.15	29
P15	66	6	Cranial Dystonia	13	86.54	38
P16	77	7	Cranial Dystonia	19	20.77	60
P17	66	9	Blepharospasm	9	83.08	42
Mean	64,44	9.50		12.38	70.31	32.27
SD	5,94	6.30		4.79	22.12	15.54

BRS  =  Blepharospasm Rating Scale; BDS  =  Blepharospasm Disability Scale; lower score indicates more severe functional impairment.

**Table 2:** Protocols in which each individual patient participated at the different time points.

Patient ID	HFS_LTP_	HFS_LTP-LTD_	HFS_LTP-LTP_	BTX 0	BTX 1	BTX 2	BTX 3
P01		X		X	X	X	X
P02			X				
P03	X	X	X				
P04		X		X	X	X	X
P05	X	X	X	X	X	X	X
P06	X	X	X	X	X	X	X
P07	X	X	X				
P08	X	X	X	X	X	X	X
P09	X	X	X	X	X	X	X
P10	X	X	X	X	X	X	X
P11	X	X	X				
P12	X	X	X				
P13	X	X	X	X	X	X	X
P14		X		X	X	X	X
P15		X		X	X	X	X
P16	X	X	X				
							
Total number	N = 11	N = 15	N = 12	N = 10	N = 10	N = 10	N = 10

LTP  =  long term potentiation; LTD  =  long term depression; LTP-LTD, LTP-LTP  =  combination of two interventions; BTX 0 =  baseline, BTX 1, BTX 2, BTX 3 = 1, 2, 4 weeks after BTX injection.

**Table 3:** Protocols in which each individual control participated at the different time points.

Control ID	Age (ys)	HFS_LTD_	HFS_LTP_	HFS_LTP-LTD_	HFS_LTP-LTP_	HFS_NO_
K01	23	X	X	X	X	X
K02	24	X	X	X	X	X
K03	65	X	X	X	X	X
K04	52	X	X	X	X	X
K05	50	X	X		X	
K06	60	X	X	X	X	X
K07	48	X	X	X	X	X
K08	51	X	X		X	X
K09	51	X	X	X	X	X
K10	54	X	X	X	X	
K11	63	X	X	X	X	X
K12	58	X	X	X	X	X
Total number		N = 12	N = 12	N = 10	N = 12	N = 10
Mean	49.92					
SD	13.45					

LTP  =  long term potentiation; LTD  =  long term depression; LTP-LTD, LTP-LTP  =  combination of two interventions.

### Interventional protocols: HFS conditioning of the electrically evoked R2 response

**Figure 1:**
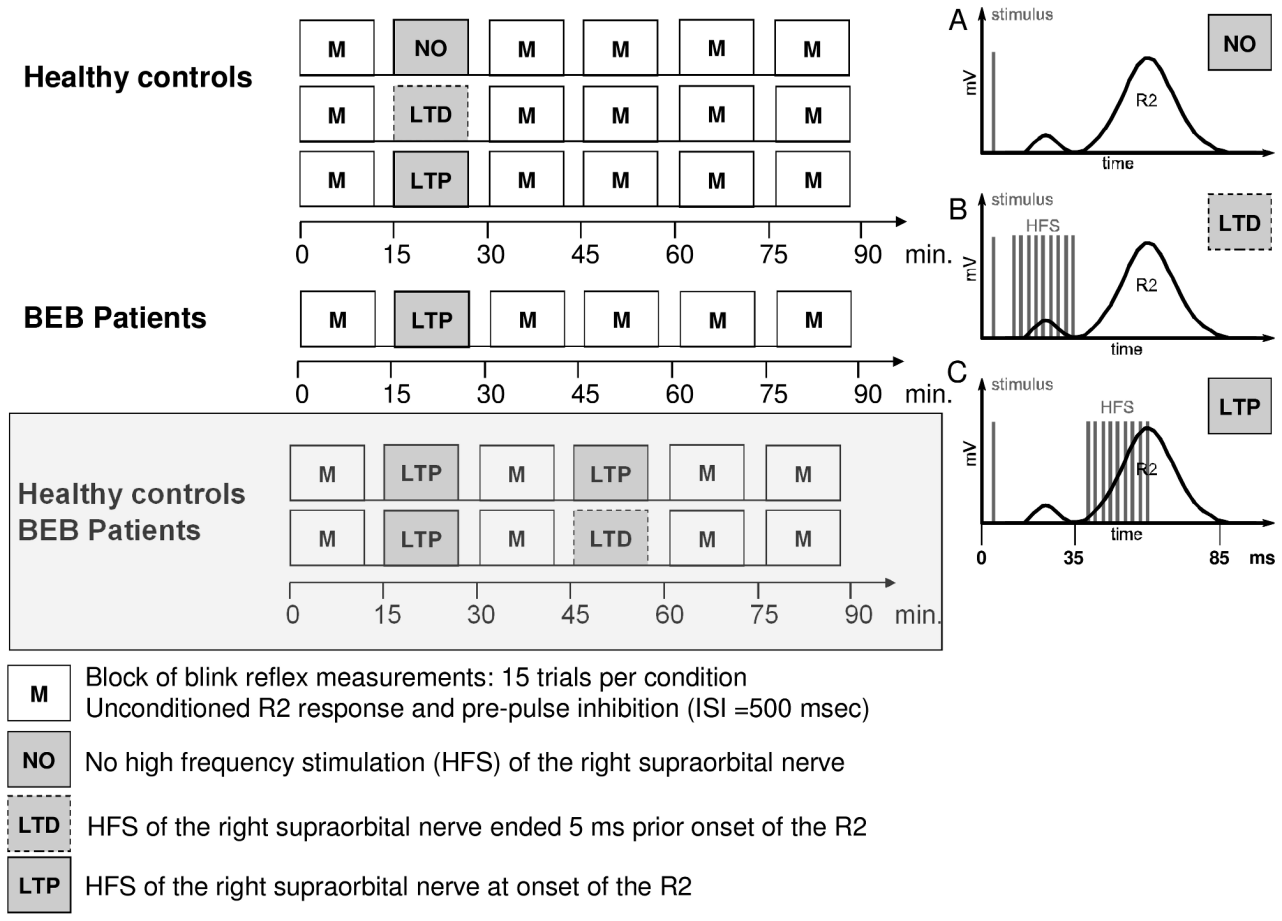
Assessing the dynamic modification range of LTP-LTD-like plasticity. Different protocols of high frequency stimulation (HFS) are presented. In controls and patients HFS_LTP_, HFS_LTP-LTD_, HFS_LTP-LTP_ protocols were investigated. The HFS_LTD_ and HFS_NO_ protocol in controls served as control condition. In the right panel the timing for each intervention is illustrated. **Panel A:** The R1 and R2 answers are illustrated. In the non-intervention protocol, no high frequency stimulation was applied. **Panel B:** The high frequency stimulation ended 5 ms before the expected R2 response to induce LTD like effects. **Panel C:** The HFS started with the onset of the R2 answer to induce LTP like plasticity.

**Figure 2:**
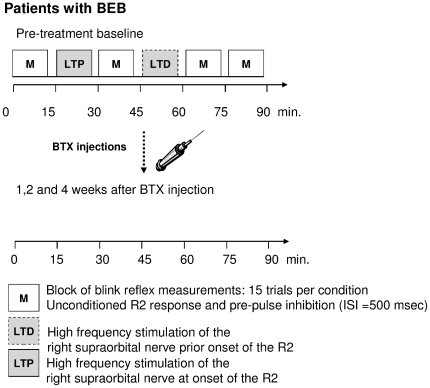
Probing the influence of BTX injections on homeostatic-like plasticity. HFS_LTP-LTD_ before, one, two and four weeks after Botulinum toxin (BTX) treatment was investigated in patients with blepharospasm.

HFS was repeatedly given directly at the onset of the R2 component of electrically evoked blinks to induce LTP like plasticity (HFS_LTP_) in patients and controls. Separate control experiments were conducted only in controls and included HFS shortly before the onset of the R2 component (HFS_LTD_) and a non-intervention protocol (HFS_NO_). We further evaluated homeostatic control by combining two facilitory protocols (HFS_LTP_ followed by HFS_LTP_) and facilitory with inhibitory interventions (HFS_LTP_ followed by HFS_LTD_) (Fig. 1). The influence of BTX on homeostatic control was investigated in the (HFS_LTP_ followed by HFS_LTD_) protocol in BEB patients (Fig. 2). For detailed descriptions see “Experimental Procedure”.

**Figure 3:**
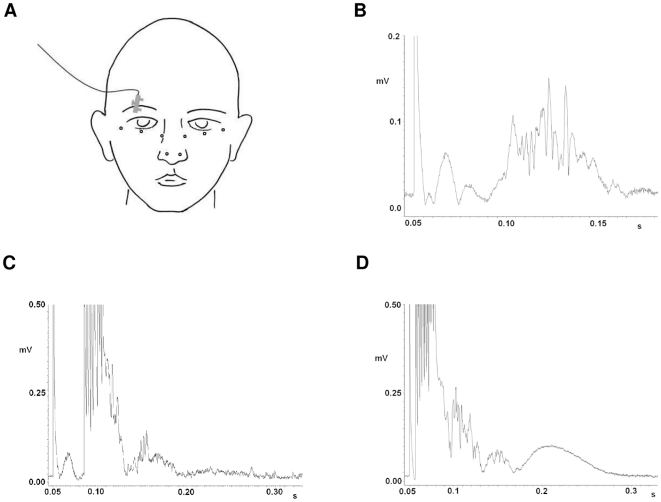
Electrode placement and examples of the R2 response. **Panel A:** Placement of the electrodes around the orbicularis oculi and nasalis muscles. The right supraorbital nerve was stimulated. **Panel B:** Example of ipsilateral R1 and R2 responses after single pulse stimulation. **Panel C:** Example of HFS_LTP_ protocol. High frequency stimulation was given at the onset of the R2 response to induce LTP-like effects. **Panel D:** Example of HFS_LTD_ protocol. Here, HFS was applied before the onset of the R2 response to induce LTD-like effects.

Electrical stimulation of the right supraorbital nerve was performed with a peripheral nerve stimulator and silver/silver chloride disc surface electrodes (DS7A Stimulator, Digitimer Ltd., Welwyn Garden City, Hertz, UK). The cathode was placed over the right supraorbital foramen and the anode 2 cm above the foramen (Fig. 3). Electrical stimuli had a square-wave configuration with a pulse width of 200 µs.

In each experiment the minimum intensity for a 200 µs stimulus required to produce a reliable R2 blink reflex component was determined (motor threshold). The motor threshold was measured twice, at baseline, and just before the last measurement. The same procedure was applied for the sensory threshold, which was defined as the minimum intensity needed to just notice the stimulation impulse. The stimulus intensity was set at two times the motor threshold (2TR2) to evoke a consistent R2 component. All experiments used the interventional HFS protocols introduced by Mao and Evinger [13]. The associative HFS protocol was given in three blocks separated by five minutes. During each block, four trains of HFS separated by 10 sec were applied to the right supraorbital nerve (Fig. 1 and 2). Each train consisted of short bursts with nine stimuli given at a rate of 400 Hz (20 ms) and an intensity of 2TR2. HFS was given at the onset of the electrically evoked R2 response to induce LTP-like effects (Fig. 3C), while HFS preceded the electrically evoked R2 response by 5 ms to produce LTD-like plasticity (Fig. 3D). We recorded the average of ten R2 responses in each participant, and individually determined the exact onset of the R2 responses to optimize the timing of the HFS. The timing for LTD inducing high frequency stimulation was calculated such that it ended exactly 5 ms before the expected R2 response [13]. The stimulation paradigm is referred to as “associative HFS” as each of the short HFS bursts (20 ms) is associated with a single electrical pulse to the supraorbital nerve preceding the HFS burst. The precise timing within these repeated pairings then determines whether the HFS takes place during or directly before the evoked R2 response of the blink reflex and thereby modulates the direction of the induced plasticity in parallel to the rules of spike timing-dependent plasticity (STDP). The associative HFS protocol in total (sum of all repeated applications of single-pulse evoked R2 responses and HFS burst) lasted 15 minutes, whereas a single HFS burst took only 20 ms. Our high frequency stimulation protocols share the same rational as the paired associative protocols that combine electrical nerve stimulation with contralateral transcranial magnetic stimulation referred to in the responses below [23], [24]. All these in vivo protocols in humans are inspired by former work about spike timing dependent plasticity on slice preparations [25]

### Measurement of the R2 response

Before and after HFS, the excitability of the blink reflex circuit was assessed in blocks of measurements (Fig. 1 and 2). In each block, 15 responses per stimulation condition were recorded followed by a pause of 5 min between each block of measurement. For measurements of the R2 response, the intensity of the supraorbital electrical stimulus was adjusted as described for the HFS treatment (2TR2). In addition to the unconditioned R2 response elicited by a single test stimulus, paired-pulse inhibition was assessed by conditioning the test stimulus with a pre-pulse of equal intensity and pulse width given 0.5 sec before [19]. For both single and paired-pulse stimulation, the inter-trial intervals were jittered (10±2 sec) in a pseudorandomized order, therefore in contrast to Quartarone et al. [14], each stimulus was triggered automatically after a predefined time interval and not manually (personal communication).

EMG activity was recorded from bilateral orbicularis oculi muscles, and as the reference from the nasalis muscle. The EMG signal was amplified by 1000 and bandpass filtered (20 Hz to 2 KHz; D360 amplifier, Digitimer Ltd., Welwyn Garden City, Hertz, UK) and stored at a sampling rate of 5 KHz on a personal computer for off-line analysis (Signal Software, Cambridge Electronic Design, Cambridge, UK). The area of the R2 response elicited by the test pulse was calculated for each condition by integrating the rectified EMG activity of the orbicularis oculi muscles using Signal Software. The onset of the R2-response was defined as a more than twofold increase in EMG activity relative to baseline level. For paired-pulse measurements, the R2 response to the test pulse was expressed as percentage change from the R2 response to the single-pulse (R2 response after paired-pulse stimulation/R2 response after single-pulse stimulation * 100–100). This enabled us to probe the excitability of inhibitory interneuronal pathways controlling the R2 response.

### Experimental Procedures

HFS_LTP_ was applied in both groups at the onset of the R2 response to induce LTP-like effects and we expected facilitation of the R2 response. Additional measurements were conducted in controls: First, HFS preceded the electrically evoked R2 response and ended 5 ms before the expected R2 response occurred to induce LTD-like plasticity (HFS_LTD_) with suppression of the R2 amplitude. After analyzing the individual onset of the R2 response in each subject, the timing for the high frequency stimulation was calculated such that it ended exactly 5 ms before the expected R2 response. Second, a “non-intervention protocol” in controls (HFS_NO_) included a pause for the time needed to apply HFS intervention.

In both, patients and controls, HFS_LTP_ was followed by either another HFS_LTP_ (HFS_LTP-LTP_) or HFS_LTD_ (HFS_LTP-LTD_). In patients, we expected that inhibitory HFS would induce less suppression of the R2 response after pre-conditioning with HFS_LTP_ (indicating an impaired depotentiation), while facilitation might be enhanced after pre-conditioning with the HFS_LTP_ protocol. All experimental sessions were performed in a counterbalanced order at least three days apart to exclude any carry-over effects.

In patients we studied the influence of BTX treatment on homeostatic plasticity modulated by the HFS_LTP-LTD_ protocol before and one, two, and four weeks after BTX injections (Fig. 2).

### Statistical Analysis

Changes in single pulse R2 responses and paired-pulse inhibition were assessed as dependent variables in separate analyses as follows: First, a three-factorial ANOVA was computed comprising the *time of measurement* (baseline, 30, 60, 75 min) and *protocol* (HFS_LTP_, HFS_LTP-LTD_, HFS_LTP-LTP_) as within-subject factor, and *group* (patients vs. controls) as between-subjects factor. As two additional protocols were conducted in controls only, we additionally computed a separate two-factorial ANOVA for *time of measurement* (baseline, 30, 60, 75 min) and *protocol* (HFS_LTP_, HFS_LTP-LTD_, HFS_LTP-LTP_, HFS_LTD_, HFS_NO_). In patients, baseline differences in clinical scores (BRS, BDS number of blinks) between protocols (HFS_LTP_, HFS_LTP-LTD_, HFS_LTP-LTP_) were evaluated by separate one-factorial ANOVAs. The effects of BTX treatment after single pulse stimulation were tested with two-factorial ANOVAs with the within-subject factors *time of measurement* (baseline, 30, 60, 75 min) and *week of measurement* (baseline, week 1, week 2, week 4). Changes in the clinical scores of patients due to BTX treatment were evaluated by one-factorial ANOVAs for the within-subject factor *time* (before BTX treatment, and 1, 2, 4 weeks after the injection), separately for the BRS, BDS and the total number of blinks.

If necessary, Greenhouse-Geisser method was used to correct for non-sphericity. Conditional on the respective significant *F* value, post-hoc paired (within-subject factor) or independent (between-subject factor) t-tests were used to explore the direction of main effects or the patterns of interaction between experimental factors. A p-value of 0.05 or less was considered significant. The results are reported as mean ± SD.

Further, the relation between percent changes of motor and sensory thresholds over time (from baseline to 60 min) and associated percent changes in the R2 response (from baseline to 60 min) were evaluated with the Pearson correlation pooling across protocols and groups (N = 89).

## Results

### Clinical results in patients

The BRS, BDS and number of blinks, measured before each interventional protocol, revealed no significant differences between protocols (BRS p>0.3; BDS p>0.1, blink rate p>0.3). In the HFS_LTP_ protocol, the mean BRS was 11.55±4.85 points, the BDS 76.78±13.00%, and the blink rate 31.45±13.99 blinks before and 30.56±18.04 blinks after the session (p>0.6). In the HFS_LTP-LTD_ protocol the BRS showed a score of 11.54±4.81 points, the BDS of 72.43±23.00%, and the blink rate of 36.79±20.49 blinks before and of 27.75±15.93 blinks after the session (p>0.7). The HFS_LTP-LTP_ intervention revealed the following clinical data: BRS 11.75±4.79 points, BDS 75.87±17.86%, blink rate 35.42±22.42 blinks before and 34.33±22.33 blinks after the session (p>0.7).

### R2 response of the blink reflex due to single pulse stimulation

**Figure 4:**
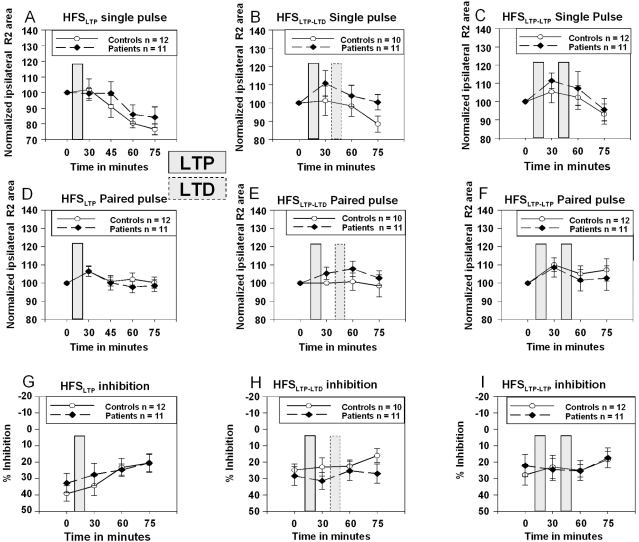
Presentation of normalized data for HFS_LTP_, HFS_LTP-LTD_, HFS_LTP-LTP_ protocols. **Panels A–C** show the normalized area under the curve of the ipsilateral R2 response after single pulse stimulation. HFS_LTP_, HFS_LTP-LTD_, HFS_LTP-LTP_ were applied in patients and controls. In **panels D, E, F** the ipsilateral R2 area under the curve after paired-pulse stimulation are depicted. In **panels G,H,I** the % inhibition is shown. Here, inhibition was determined as percent change from the R2 response after paired-pulse stimulation relative to single pulse stimulation. Single pulse stimulation revealed a nonspecific decrease of the R2 response in all protocols and in both groups. After paired-pulse inhibition no changes were found, because the decrease of the R2 response was accompanied by a decrease of % inhibition (higher values indicate stronger inhibition). R2 area under the curve is given in µV/sec. Mean and standard error are shown. Open circles indicate controls, black diamonds patients.

The stimulation intensity used for the right supraorbital nerve was on average 7.4±1.9 mA in patients, and 7.5±2.2 mA in controls. The three-factorial ANOVA revealed no main effects of *protocol* (p>0.1) or *group* (p>0.5), and no interaction (p>0.1), but a main effect of *time* (F_1.75, 33.15_ = 8.62; p = 0.001; Fig. 4a–c). The R2 responses decreased from baseline to 75 min (T_20_ = 3.71; p<0.001), 30 to 60 min (T_20_ = 2.70; p = 0.014), 30 to 75 min (T_20_ = 3.60; p = 0.002) and finally 60 to 75 min (T_20_ = 3.04.; p = 0.006). The two-factorial AVOVA for controls confirmed the non-specific decrease of the R2 response over time (F_1.56, 12.44_ = 9.9; p = 0.004; Fig. 4a–c, 5a). The *time* effect emerged between baseline and 60 min (T_8_ = 3.32; p = 0.010), baseline and 75 min (T_8_ = 3.92; p = 0.004), 30 and 60 (T_8_ = 3.18; p = 0.013), 30 and 75 min (T_8_ = 3.14; p = 0.014) and 60 and 75 min (T_8_ = 2.45; p = 0.040).

**Figure 5:**
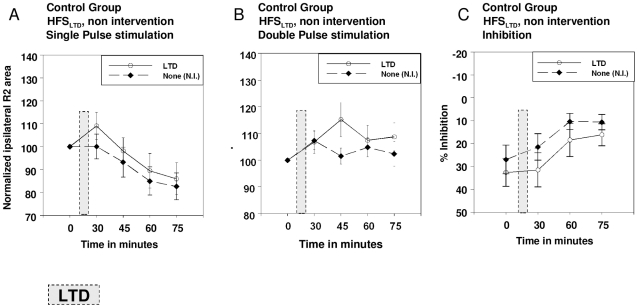
Presentation of normalized data for HFS_LTD_ and HFS_non-intervention_ protocols in controls. **Panel A** presents the results after single pulse, **panel B** after paired-pulse stimulation, and **panel C** % inhibition (higher values indicate stronger inhibition). R2 area under the curve is given in µV/sec. Mean and standard errors are shown. Circles indicate the HFS_LTD_, black diamonds the non-intervention protocol.

**Figure 6:**
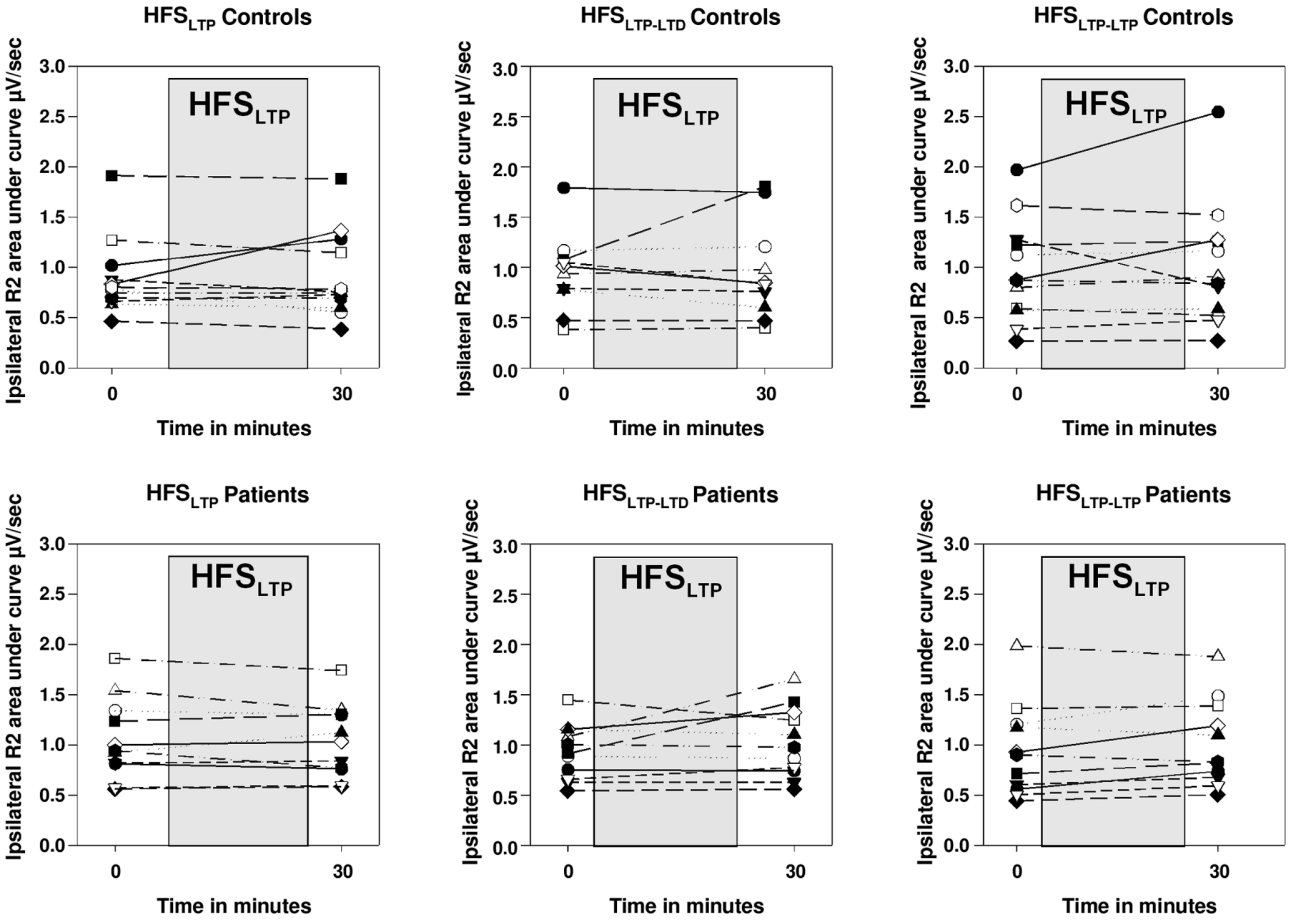
R2 response of each individual subject at baseline and after HFS_LTP_. Individual results of each subject before and immediately after HFS_LTP_ for each protocol starting with HFS_LTP_ (i.e. HFS_LTP_, HFS_LTP-LTD_, HFS_LTP-LTP_) are presented.

**Figure 7:**
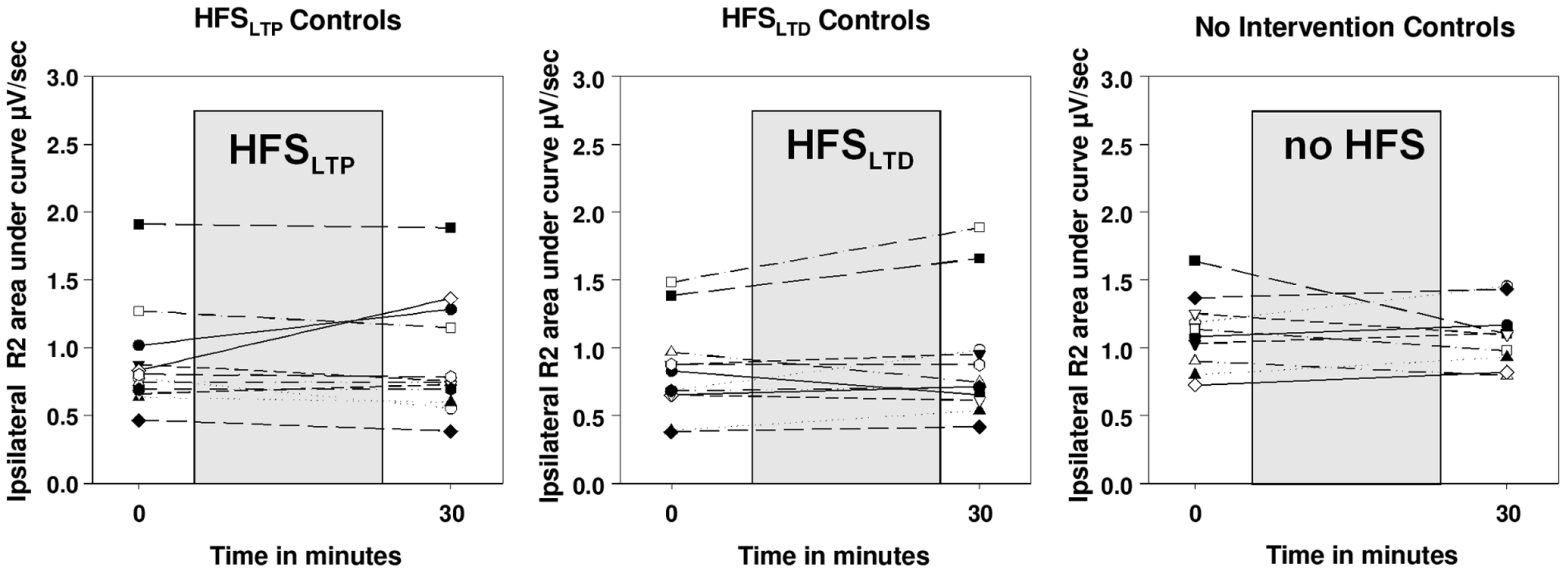
R2 response of each control person at baseline and after HFS_LTP_, HFS_LTD_, and HFS_non-intervention_. In analogy to Figure 6, individual results of each control subject before and immediately after HFS for the HFS_LTP_, HFS_LTD_ and non-intervention protocol are presented.

We were interested to explore the inter-subject variability in the acute changes of the R2 response after the first HFS_LTP_ intervention. When plotting the immediate change in R2 response after HFS_LTP_ in the three interventional sessions, some subjects showed a marked increase in one experimental session, but this R2 facilitation could not be replicated in the other sessions (Fig. 6). In healthy controls, inter-subject variability of changes in R2 area after HFS_LTP_ was within the range of spontaneous fluctuations in the R2 response observed in the HFS_NO_ session (Fig. 7). To further increase the sensitivity to detect any facilitatory effects of the HFS_LTP_ protocol we reduced the variability by calculating the percent change between baseline and the 30 min measurement averaged across HFS_LTP_, HFS_LTP-LTD_ and HFS_LTP-LTP_ protocols. A one-sided one-sample t-test detected a mild facilitatory effect of 7% only in patients (T_10_ = 2.46; p = 0.017), but not in controls (p>0.4). A two-tailed independent sample t-test between patients and controls, however, showed no significant difference between the two groups (p>0.6).

Further, a possible influence of the motor threshold on the R2 response size was assessed. We calculated the correlation between changes in motor thresholds and changes in the R2 response from baseline to 60 min and found a very weak but statistically significant negative correlation (r = −0.280; p = 0.008). A higher motor threshold was accompanied by a decreased R2 response, while changes in sensory threshold showed no correlation with the R2 response (r = −0.063; p>0.5).

### Inhibition of the R2 response due to paired-pulse stimulation

A significant % inhibition of the R2 response was detected in patients and controls in all protocols at all time points (separate one-sided one-sample t-tests; p<0.014; Fig. 4d–f; Fig. 5b). The three-factorial ANOVA revealed no main effects of *protocol* (p>0.1) or *group* (p>0.3), but a main effect of *time* (F_1.75, 37.29_ = 6.62; p = 0.003) and an additional interaction between time* protocol (F_6, 108_ = 2.43; p = 0.03) occurred. A general decrease in % inhibition (averaged across protocols) occurred from baseline to 60 min (T_20_ = 2.16; p = 0.043), baseline to 75 min (T_20_ = 4.64; p<0.001), 30 to 75 min (T_20_ = 2.76; p = 0.012) and finally 60 to 75 min (T_20_ = 2.59.; p = 0.017). Based on the significant interaction, post hoc t-tests were further calculated separately for all protocols revealing the following decreases of % inhibition over time; HFS_LTP_ protocol: from baseline to 30 min (T_20_ = 2.30; p = 0.007), baseline to 60 min (T_20_ = 2.26; p = 0.035), baseline to 75 min (T_20_ = 3.94; p = 0.001); HFS_LTP-LTP_ protocol: baseline to 75 min (T_20_ = 2.17; p = 0.042). No significant changes in % inhibition occurred in the HFS_LTP-LTD_ protocol (Fig. 4g–i).

The same finding was confirmed in the separate two-factorial analysis for controls, in which a *time* (F_3, 21_ = 16.29; p<0.000) and time * protocol interaction (F_12, 84_ = 1.92; p = 0.043) effect was identified. One control subject had to be excluded from this analysis (N = 8), as paired-pulse stimulation data were incomplete for the 60 minutes measurement in the HFS_LTP-LTD_ protocol. Inhibition decreased on average (across protocols) from baseline to 60 (T_7_ = 9.50; p<0.001), baseline to 75 (T_7_ = 5.91; p = 0.001), 30 to 60 (T_7_ = 2.98; p = 0.021) and 30 to 75 (T_7_ = −2.76; p = 0.028) min. Exploring the interaction effect in controls, separate post-hoc paired t-test for each protocol retrieved the following reductions in % inhibition; HFS_LTP_ protocol: Baseline to 30 (T_7_ = 3.22; p = 0.015), baseline to 60 (T_7_ = 5.37; p = 0.001), baseline to 75 (T_7_ = 9.14; p<0.001), and 30 to 75 (T_7_ = 4.41; p = 0.003) min (Fig. 4g); HFS_LTD_ protocol: Baseline to 60 (T_7_ = 2.37; p = 0.049), baseline to 75 (T_7_ = 3.66; p = 0.008), 30 to 60 (T_7_ = 3.15; p = 0.016) and 30 to 75 (T_7_ = 3.23; p = 0.015) min (Fig. 5c); HFS_NO_ protocol: Baseline to 75 (T_7_ = 2.66; p = 0.032) min (Fig. 5c). The HFS _LTP-LTD_ and HFS _LTP-LTP_ protocols showed no significant changes in % inhibition (Fig. 4h,i). Since a decrease of the R2 response was accompanied by a respective decrease of % inhibition, the actual R2 responses to the test pulses of paired-pulse stimulation varied only slightly during the time course (Fig. 4d–f; 5b). There was no correlation between the change of the sensory or motor thresholds and the change of inhibition over time.

### Results in patients before and after BTX treatment

#### Clinical results in patients before and after BTX treatment

BTX treatment caused no significant changes in the BRS (baseline: 13±5.2 points; week 1: 13±4.0 points; week 2: 12.1±4.2 points; week 4: 10.7±4.6 points) or in the BDS (baseline: 67.7±27.2%; week 1: 64.6±25.2%; week 2: 63.8±26.3%; week 4: 71.9±15.7%) but a trend for a decrease in blink rate (F _3; 21_ = 2.52; p = 0.085; baseline: 38.6±18.6; week 1: 37.7±19.1; week 2: 26.5±16.5; week 4: 23.8±12.5).

#### HFS_LTP-LTD_ intervention before and after Botulinum Toxin Treatment

**Figure 8:**
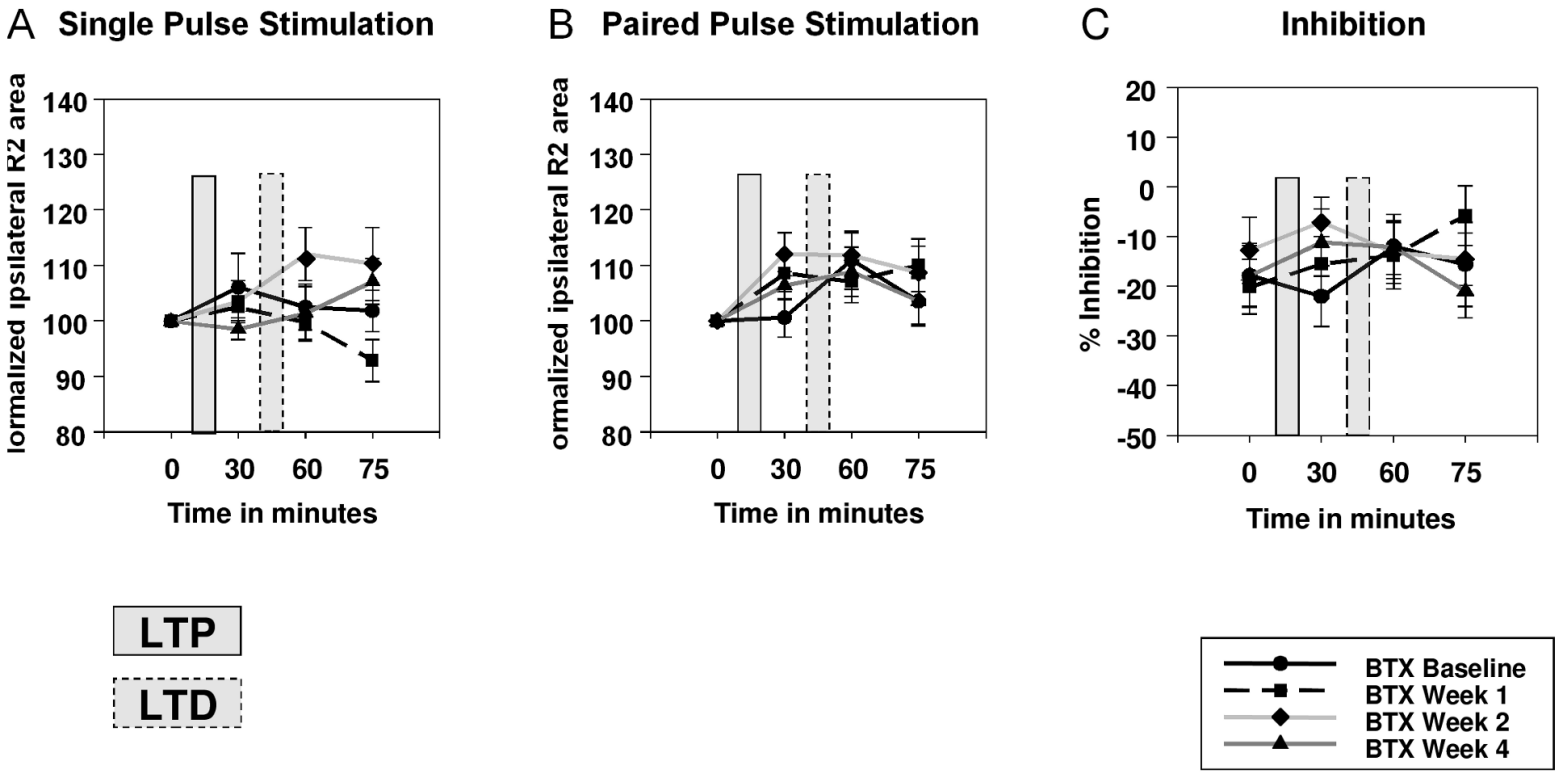
HFS_LTP-LTD_ before and after Botulinum Toxin Treatment. Results of normalized data for HFS before and after Botulinum toxin (BTX) treatment in BEB patients are shown for HFS_LTP-LTD_. **Panel A**: Single pulse; **Panel B**: Paired-pulse. BTX had no effect on the HFS_LTP-LTD_ protocol. Black line with circle  =  baseline; black, dashed line with square  =  one week after BTX injection; light grey line with diamond  =  two weeks after BTX; dark grey line with triangle  =  four weeks after BTX. R2 area under the curve is given in µV/sec. % inhibition is relative to single pulse measurements.

Paired-pulse inhibition per se was preserved after BTX treatment (one-sample t-tests; baseline: T_9_ = 2.79; p = 0.021; week 1: T_9_ = 3.67; p = 0.005; week 2: T_9_ = 1.92; p = 0.087; week 4: T_9_ = 2.93; p = 0.017). However, BTX did not either alter the R2 response or % inhibition or had any effect on the time course within the HFS_LTP-LTD_ protocol (Fig. 8).

## Discussion

In this study, we used associative HFS of the supraorbital nerve in an attempt to induce LTP- and LTD-like plasticity and study their homeostatic interactions in patients with BEB and healthy controls. HFS was given before (HFS_LTD_) or during (HFS_LTP_) the occurrence of an electrically evoked R2 response of the trigeminal blink reflex. Contrary to our expectation the conditioning effects of HFS on the size of the R2 responses did not differ between patients with BEB compared to controls. In both groups, the three HFS protocols (HFS_LTP_, HFS_LTP-LTD_, HFS_LTP-LTP_) produced a non-specific decrease of the R2 response after 60 and 75 min compared to pre-interventional baseline. These changes were not specifically related to the various associative HFS protocols, as healthy controls also showed a similar decrease in R2 during the experiment when no associative HFS was applied. Overall, the R2 changes induced by the first HFS_LTP_ protocol varied considerably both across subjects and sessions.

Our results contrast with the findings of previous studies [13], [14], where LTP-like effects were consistently observed after associative HFS at the onset of the R2 response. Furthermore, patients with BEB showed an excessive LTP-like facilitation of the R2 response after HFS_LTP_ [14] which was not evident in our analyses. In accordance with the group analysis there was considerable intra-subject variability across protocols applied as shown in figures 6 and 7. After reducing the variability by pooling the baseline and post- HFS_LTP_ measurements of the three sessions in which we applied HFS_LTP_ (i.e., HFS_LTP_, HFS_LTP-LTD_, and HFS_LTP-LTP_ sessions), there was a small (7%) increase in R2 area in BEB patients 30 min after the HFS_LTP_ intervention, but not in healthy controls, which might be attributed to higher excitability in patients compared to controls.

Since we used exactly the HFS procedures as described by Mao and Evinger and Quartarone et al. [13], [14], differences in the plasticity inducing procedures may not account for the lack of positive findings in the present study. Most importantly, the relative timing between the HFS burst and the electrically evoked R2 response in the HFS_LTP_ and HFS_LTD_ protocols was identical. Of note, we also used the same Signal software configuration file which was kindly provided by A. Quartarone when setting up the protocol. There are, however, some differences in the experimental procedures that need to be discussed. To facilitate an in-depth comparison of our results with previous work, the methodological details of the three studies are summarized in table 4.

**Table 4:** Differences in the methods used between the experiments.

Techniques	Mao and Evinger [13]	Quartarone et al. [14]	Present study
Number of subjects	5 subjects per protocol	16 patients11 controls	16 patients12 controls
AgeYears ± SD	Not given	Controls 59±9Patients 64±12	Controls 50±14Patients 64±10
Protocols applied	HFS_LTP_, HFS_LTD_, control condition	HFS_LTP_	HFS_LTP_, HFS_LTP-LTD_, HFS_LTP-LTP_, HFS_LTD_, HFS_NO_
Pulse width	170 µsec	200 µsec	200 µsec
Threshold	Minimum intensity for reliable R2 response	Minimum intensity for R2 ≥ 50 µV	Minimum intensity for reliable R2 response
Relative stimulus intensity	2 times the threshold to evoke reliable R2 response (2TR2)	2 times the threshold to evoke reliable R2 response (2TR2)	2 times the threshold to evoke reliable R2 response (2TR2)
Absolute stimulus intensity	Not given	11.5±5.3 mA	7.5±2.3 mA
Interstimulus intervals	Pairs of stimuli with 7.5 sec interstimulus interval alternately to left and right side every 25±5 sec	Manually triggered	Automatically jittered between 10±2 sec
Side of stimulation	Right and left	right	right
Dependant variable used for statistical analysis	Normalized R2 amplitudes, of the treated minus untreated side	Integrated area of rectified R2 response	Integrated area of rectified R2 response
Duration of experiment	60 minutes	60 minutes	75 minutes
Single vs. paired pulse	Single pulse, number not given	20 Single pulse	15 Single and 15 Paired Pulse of SO

We used a lower stimulus current than Quartarone et al. [14] to avoid C-fiber activation and pain [26] because we were concerned that this might adversely affect the induction of LTP-like or LTD-like plasticity [27]. On the other hand, it is possible that a certain threshold intensity has to be exceeded to induce LTP or LTD like effects with associative HFS. Increasing stimulus intensities activates more motor units and influences the R2 amplitude and paired-pulse inhibition. While some blink reflex studies applied stimuli with a current of 16–26 mA [28], others applied only 3–10 mA [19]. Intensities below 5 mA and above 26 mA are problematic as it becomes difficult to detect differences between BEB patients and controls [29]. Within the intensity range used, we found no correlation between the intensity and the R2 response. Therefore, we consider the moderate difference in stimulus intensity between our study and Quartarone et al. [14] to be of limited importance. Yet, this aspect needs to be evaluated in more detail.

To avoid habituation effects, it is crucial to use appropriately long interstimulus intervals [30]. The interval we chose was jittered at 10±2 sec which was shorter than the interstimulus intervals used in the study by Mao and Evinger [13]. Since we combined HFS_LTP_ with HFS_LTD_ and two HFS_LTP_ protocols, our experiment lasted longer than the experiments by Mao and Evinger [13] and Quartarone et al. [14]. The gradual decrease in R2 area that we observed during the course of all experimental conditions might represent a habituation effect of continuous measurements on electrical excitability of the R2 response after 60 and 75 min. Indeed, habituation has been described for the trigeminal blink reflex as slow (1 Hz) repetitive stimulation is sufficient to suppress the R2 component [19]. We propose that the longer duration of the experimental procedure might have unmasked habituation effects that were missed in previous studies using associative HFS due to shorter experimental procedures.

HFS, that precedes the electrically evoked R2 response to produce LTD-like plasticity, has so far only been applied by Mao and Evinger in five healthy subjects. Paired associative stimulation (PAS) repeatedly pairs electrical stimulation of the median nerve at the wrist and transcranial magnetic stimulation (TMS) of the contralateral motor cortex with a specific interstimulus interval. It is a well documented associative stimulation protocol to noninvasively induce plasticity in the human motor cortex [23], [24]. TMS studies have shown that practice-dependent plasticity declines as a function of age in subjects older than 50 years [31]. Further, a reduction of the PAS-induced plasticity of the primary motor cortex in elderly subjects has been documented [12], [32], [33]. Possibly, it is more difficult to induce LTD-like plasticity after HFS_LTD_ protocol in the blink reflex circuit in older subjects. An attenuating age effect on HFS induced trigeminal plasticity might be an important factor accounting for the inefficiency of associative HFS in our study.

In contrast to previous studies, we examined the effects of associative HFS on both single-pulse excitability and paired-pulse inhibition of the R2 response. Single pulse and paired-pulse stimulation was intermingled in a pseudo-randomized fashion during the blocks of measurements. This also increased the total number of electrical stimuli applied to the supraorbital nerve that were applied before the first HFS protocol.

Repetitive transcranial stimulation (rTMS) of the same intensity applied to the primary motor cortex can induce a modulation of cortical excitability which ranges from inhibition to facilitation depending on stimulation variables. In a recent study, PAS of the contralateral primary motor cortex failed to induce bi-directional shifts in corticospinal excitability when PAS was preceded by 0.1 Hz rTMS of the motor cortex [34]. The priming 0.1 Hz rTMS protocol presumably induced lasting increases in the excitability of intracortical inhibitory circuits in the motor cortex. Therefore, it was hypothesized that 0.1 Hz rTMS reduced the susceptibility of the stimulated motor cortex to the conditioning effects of subsequent PAS by strengthening intracortical inhibition. The increase in intracortical inhibition after low-frequency rTMS might have thus interfered with associative stimulation and prevented the induction of spike timing-dependent plasticity in the motor cortex [34], [35]. Although we consider this unlikely, the application of 15 single-pulse and 15 paired-pulse stimuli at approximately 0.1 Hz might have had an “occlusion effect” interfering with the efficacy of subsequent associative HFS protocols to induce spike-timing dependent plasticity. If so, the number of R2 measurements should be minimized in future studies to minimize any occluding effects on associative plasticity.

The relative magnitude of paired-pulse inhibition of the R2-response decreased significantly after HFS_LTP_, HFS_LTD_ and HFS_NO_, while no significant changes were detected after applying the combination of the HFS_LTP-LTD_ and HFS_LTP-LTP_ protocols. This might be related to some homeostatic processes that regulate the excitability of inhibitory neurons mediating paired-pulse inhibition of the R2 response [28], [29]. Considering the long duration of the R2 inhibition, the influence from more distant neural structures is possible [29], [30], [36]. In fact, several brain regions seem to exert control over the excitability of motoneurons and interneurons in the R2 circuit. The basal ganglia might influence the blink reflex via direct subcortical pathways [19]. Since we observed a decrease of the R2 response after single pulse stimulation, but a decrease of inhibition after 60 and 75 min, the decrease in inhibition could be a form of homeostatic regulation mediated through descending inputs to prevent further down-regulation of the R2 response to preserve the protection of this adverse-effects reflex. This speculation needs to be further investigated.

In contrast to the study by Quartarone et al. [14], BTX did not modulate the recovery curves of the blink reflex as assessed with paired-pulse stimulation, showing that BTX has little effect on the enhanced excitability of brainstem interneurons in patients with BSP. In our study, the HFS_LTP-LTD_ protocol was not altered by BTX treatment in our BEB patients.

In summary, our results differ from the findings of Mao and Evinger's and Quartarone et al. There are slight differences in the methods used. It is conceivable that the duration of our protocol produced a habituation effect that was not seen before in the shorter protocols used. Further we cannot exclude that repeated paired-pulse stimulation caused lasting inhibition and thus blocked the ability of associative HFS to induce spike-time dependent like plasticity in human blink reflex circuit. Our study also shows that the method is not reliable to investigate homeostatic properties of the blink reflex recovery cycle.
